# Investigation of the Mechanisms Underlying the Development and Evolution of the Cerebral Cortex Using Gyrencephalic Ferrets

**DOI:** 10.3389/fcell.2022.847159

**Published:** 2022-03-21

**Authors:** Yohei Shinmyo, Toshihide Hamabe-Horiike, Kengo Saito, Hiroshi Kawasaki

**Affiliations:** Department of Medical Neuroscience, Graduate School of Medical Sciences, Kanazawa University, Kanazawa, Japan

**Keywords:** cerebral cortex, development, evolution, ferret, gyrification

## Abstract

The mammalian cerebral cortex has changed significantly during evolution. As a result of the increase in the number of neurons and glial cells in the cerebral cortex, its size has markedly expanded. Moreover, folds, called gyri and sulci, appeared on its surface, and its neuronal circuits have become much more complicated. Although these changes during evolution are considered to have been crucial for the acquisition of higher brain functions, the mechanisms underlying the development and evolution of the cerebral cortex of mammals are still unclear. This is, at least partially, because it is difficult to investigate these mechanisms using mice only. Therefore, genetic manipulation techniques for the cerebral cortex of gyrencephalic carnivore ferrets were developed recently. Furthermore, gene knockout was achieved in the ferret cerebral cortex using the CRISPR/Cas9 system. These techniques enabled molecular investigations using the ferret cerebral cortex. In this review, we will summarize recent findings regarding the mechanisms underlying the development and evolution of the mammalian cerebral cortex, mainly focusing on research using ferrets.

## Introduction

The cerebral cortex has changed significantly in the long history of mammalian evolution (Rakic, 1995; Kriegstein et al., 2006; Molnár et al., 2006; Rakic, 2009; Fietz and Huttner, 2011; Lui et al., 2011; Zilles et al., 2013; Borrell and Götz, 2014; Florio and Huttner, 2014; Kawasaki, 2014; Sun and Hevner, 2014; Poluch and Juliano, 2015; Hatakeyama et al., 2017; Kawasaki, 2017; Llinares-Benadero and Borrell, 2019; Gilardi and Kalebic, 2021). The number of neurons and glial cells in the cerebral cortex has increased, and as a result, the cerebral cortex has markedly expanded. Along with its expansion, it developed a variety of brain structures including folds (i.e. gyri and sulci) on its surface, and its neuronal circuits increased in complexity. Although it has been proposed that the expansion of the cerebral cortex and these developed brain structures are the fundamental basis for the acquisition of higher brain functions during evolution, the mechanisms underlying the formation and evolution of these brain structures are still not fully understood.

**FIGURE 1:**
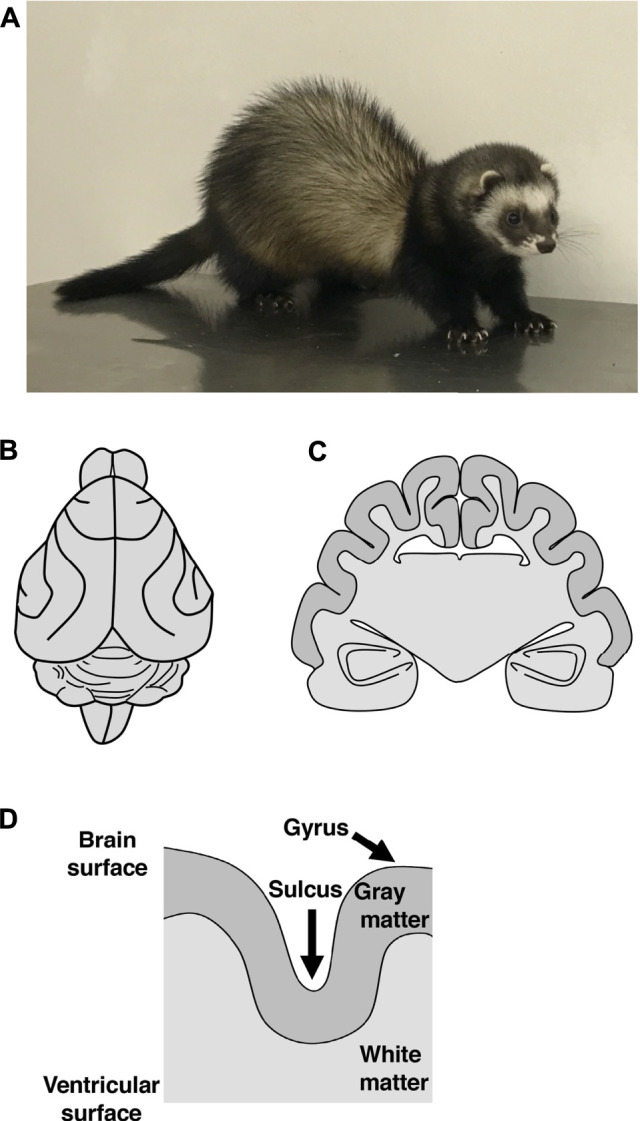
The brain structures of the ferret. **(A)** An adult ferret. A ferret is a medium-sized carnivorous mammal with a body length of about 50 cm, a weight of 1–2 kg, and an average lifespan of 6–10 years. **(B, C)** Illustrations of a dorsal view **(B)** and of a coronal cross section **(C)** of the adult ferret brain. The ferret brain has folds on the surface of the cerebral cortex. **(D)** A schema of a cross section of the cerebral cortex in gyrencephalic animals. Both the surface of gray matter and the boundary between gray matter and white matter are curved. In contrast, the surface of white matter at the lateral ventricle does not exhibit curvature. Thus, white matter is thicker at the gyrus than at the sulcus.

One reason for this is that the mouse brain, which is widely used for genetic analyses, does not have cortical folds, making it difficult to investigate the mechanisms using mice. Therefore, it seemed that genetic analyses using a well-developed cerebral cortex that shares similar properties with the human cerebral cortex would be important. For this purpose, several laboratories including ours are using the ferret (*Mustela putorius furo*) (Figure 1A), a medium-sized carnivorous mammal, because it has a relatively large and developed cerebral cortex with folds (Figures 1B,C) (Smart and McSherry, 1986; Noctor et al., 1999; Kawasaki et al., 2004; Borrell et al., 2006; Neal et al., 2007; Fietz et al., 2010; Rowell et al., 2010). Furthermore, recent progress in genetic manipulation techniques for the ferret cerebral cortex has enabled us to investigate the molecular mechanisms underlying the development and evolution of the cerebral cortex (Borrell, 2010; Kawasaki et al., 2012; Kawasaki et al., 2013; Nonaka-Kinoshita et al., 2013; Kou et al., 2015; Tsunekawa et al., 2016; Shinmyo et al., 2017; Johnson et al., 2018; Yu et al., 2019). In this review, we first introduce recent studies that used ferrets to investigate the mechanisms underlying cortical folding, the amplification of neural progenitors and the interrelationship between them. By comparing the results of these studies with findings obtained using other animal species, we further discuss common and species-specific mechanisms of cortical folding. Our recent studies demonstrated that ferrets have developed axon fiber layers in the cerebral cortex, as is the case in monkeys and humans (Saito et al., 2019). Therefore, we also discuss the formation and evolution of neuronal circuits in the mammalian cerebral cortex.

## Structural Features and Developmental Processes of Cortical Folds

One of the striking structural features of the human cerebral cortex is the large number of folds on its surface (Lui et al., 2011; Florio and Huttner, 2014; Sun and Hevner, 2014; Kawasaki, 2017; Llinares-Benadero and Borrell, 2019). Cortical folds consist of ridges called gyri and furrows called sulci (Figure 1D). It is believed that the acquisition of cortical folds during evolution led to the increase in the surface area of the cerebral cortex, allowing accommodation of many neurons within a limited capacity of the cranium. Cortical folds, therefore, are thought to be an important structural basis for the development of brain functions. Indeed, patients with abnormal cortical folds such as polymicrogyria and lissencephaly exhibit severe intellectual disability (Ross and Walsh, 2001; Fernández et al., 2016). It would therefore be important to elucidate the molecular mechanisms underlying the development and evolution of cortical folds and the pathogenesis of neurological diseases related to cortical folds.

The cerebral cortex is composed of six layers of gray matter, where neurons are concentrated, and white matter, which mainly consists of axons and myelin. Cortical folds are found in animal species with relatively large brains, such as humans, monkeys, cats and ferrets, whereas they tend to be absent in animal species with small brains, such as rats and mice. Gyrencephalic and lissencephalic refer to the presence and absence, respectively, of cortical folds. It would be intriguing to uncover the mechanisms that determine whether the cerebral cortex becomes gyrencephalic or lissencephalic.

Cortical folds have the following structural features (Borrell, 2018). Both the surface of gray matter and the boundary between gray and white matter are curved, and as a result, all six layers of gray matter are curved in accordance with cortical folds (Figure 1D). In contrast, the surface of white matter at the lateral ventricle is characterized by flatness and does not exhibit curvature (Figure 1D). Thus, white matter is thicker at gyri than at sulci. It would be important to examine whether these physiological features of cortical folds can be observed when performing experiments.

Even in animals with gyrencephalic cerebral cortices, cortical folds are not present early during development, and they appear gradually as the development of the cerebral cortex progresses. Cortical folds are formed in the embryo (i.e. before birth) in cynomolgus monkeys, while cortical folding proceeds after birth in ferrets (Welker et al., 1990). Sulci formed early during development are called the primary sulci, while those formed later during development are called the secondary and higher sulci (Welker et al., 1990). In other words, when cortical folding starts during development, the cerebral cortex exhibits simple patterns of cortical folds, having only the primary sulci. As cortical folding proceeds, the secondary and higher sulci are added, making the final patterns of cortical folds. Interestingly, the positions of the primary sulci are well-conserved between genetically identical twins, but those of the secondary and higher sulci are less-conserved, suggesting that the position of the primary sulcus is determined by genetic factors, whereas other factors are involved in the formation of the secondary and higher sulci (Lohmann et al., 1999).

## Cortical Development in Gyrencephalic Animals

**FIGURE 2:**
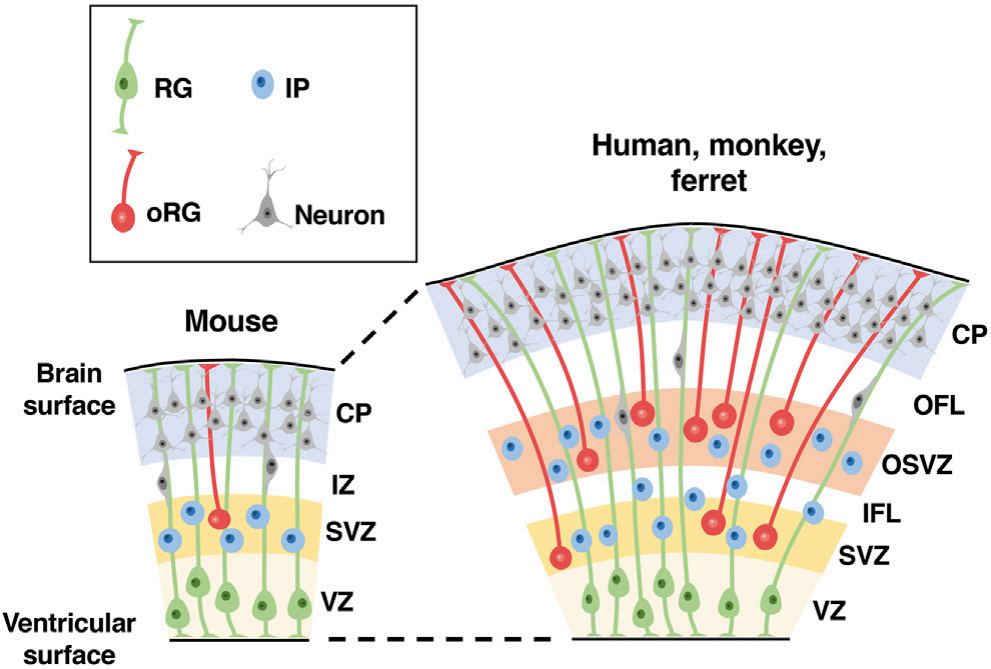
Distribution patterns of neural progenitors in the developing cerebral cortex of lissencephalic and gyrencephalic animal species. Schemas of cross sections of the developing cerebral cortices of lissencephalic mice and gyrencephalic humans, monkeys and ferrets are shown. There are three types of neural progenitors in the developing cerebral cortex: radial glial (RG) cells, intermediate progenitor (IP) cells and outer radial glial (oRG) cells. Note that compared with the lissencephalic cortex, the gyrencephalic cortex has another region of neural progenitors, the outer subventricular zone (OSVZ), in addition to the ventricular zone (VZ) and the subventricular zone (SVZ). CP, cortical plate; IZ, intermediate zone; OFL, outer fiber layer; IFL, inner fiber layer.

The cerebral cortex is formed from neuroepithelial (NE) cells surrounding the lateral ventricle (Taverna et al., 2014). NE cells give rise to radial glial cells (RG cells, also known as apical progenitors, ventricular RG cells and apical RG cells), which reside in the ventricular zone (VZ) and have bipolar radial processes between the ventricle and the pial surface of the cerebral cortex (Figure 2) (Taverna et al., 2014). The asymmetric division of RG cells produces basal progenitors including intermediate progenitor (IP) cells and outer radial glial cells (oRG cells, also known as OSVZ RG cells, basal RG cells, intermediate RG cells and translocating RG cells), which reside in the subventricular zone (SVZ) (Figure 2) (Taverna et al., 2014). In addition to the VZ and the SVZ, the developing cerebral cortex of gyrencephalic animal species, namely humans, monkeys and ferrets, has another region containing abundant oRG cells, the outer SVZ (OSVZ) (Figure 2) (Smart et al., 2002). Only a small number of oRG cells are seen in the SVZ of mice, and it is thought that an increase in oRG cells led to the production of a large number of neurons in the cerebral cortex. Cortical neurons generated from these progenitor cells migrate to the cortical plate using the radial processes of RG cells in a birth-date-dependent inside-out manner, and newly generated neurons migrate radially past existing neurons (Figure 2) (Silva et al., 2019). Thus, cortical neurons in different cortical layers are generated in a temporal sequence, such that lower-layer neurons are generated before upper-layer neurons. Migrating cortical neurons extend their axonal fibers, which compose the wiring of the brain. Gyrencephalic animals have developed axon fiber layers in the cerebral cortex (Molnár and Clowry, 2012; Saito et al., 2019), as will be mentioned later.

Because the ratio of the number of upper-layer neurons to that of lower-layer neurons is much greater in humans than in rodents (DeFelipe et al., 2002), investigations of temporal plasticity in neural progenitors in the gyrencephalic cortex have been of interest. Previous studies with heterochronic transplantations showed the presence of fate-restricted progenitors in the ferret cerebral cortex (Frantz and McConnell, 1996; Desai and McConnell, 2000). When late-stage progenitors that produce upper-layer neurons were transplanted into the cerebral cortex of younger hosts, they were not competent to generate lower-layer neurons and were restricted to producing upper-layer neurons. In contrast, recent studies using the mouse cerebral cortex revealed progenitor-type-specific differences in fate plasticity (Oberst et al., 2019). RG cells can revert their temporal identity and re-enter past neurogenic states, while IP cells are committed progenitors that lack such retrograde fate plasticity. It would be important to investigate the fate plasticity of oRG cells, which predominantly produce upper-layer neurons, in the gyrencephalic cortex (Lukaszewicz et al., 2005).

## Hypotheses on the Mechanisms Underlying Cortical Folding

Several hypotheses regarding the mechanisms of cortical folding have been proposed (Lui et al., 2011; Florio and Huttner, 2014; Kawasaki, 2014; Sun and Hevner, 2014; Kawasaki, 2017; Borrell, 2018; Llinares-Benadero and Borrell, 2019; Gilardi and Kalebic, 2021). One hypothesis is that cortical folding resulted from increased intracranial pressure because cortical folds tend to be observed in animals with larger cerebral cortices but not in those with smaller cerebral cortices (Welker et al., 1990). According to this hypothesis, as the cerebral cortex expanded in the limited volume of the cranium, increased intracranial pressure caused the cortex to fold. However, because cortical folds did not disappear when intracranial pressure was reduced experimentally, this hypothesis seems unlikely. Another hypothesis is that an abundance of oRG cells is crucial for cortical folding. Indeed, the gyrencephalic cerebral cortex has many oRG cells in the OSVZ (Figure 2) (Smart et al., 2002). Some animal species, such as marmosets, have an OSVZ, but their cerebral cortex exhibits almost no cortical folds (Kelava et al., 2012). However, it seems plausible that oRG cells in the OSVZ are important for cortical folding because the amounts of neural progenitors in the OSVZ are positively correlated with the degree of cortical folding (Reillo et al., 2011). A third hypothesis is that the ratio of the thicknesses of the superficial and deep regions in the cerebral cortex is crucial for cortical folding (Richman et al., 1975; Kriegstein et al., 2006). If superficial regions preferentially expanded relative to deep regions, it would result in an outward convex. Consistent with this hypothesis, experiments using expandable materials successfully reproduced structures similar to cortical folds of the mammalian cerebral cortex (Tallinen et al., 2016). A fourth hypothesis is that the tension created by axons connecting neighboring cortical regions produces cortical folds (Van Essen, 1997). Based on this hypothesis, axons connecting neighboring cortical regions bind these regions to each other, and the cortex between them protrudes outward. Finally, because the morphology and gene expression patterns of neural progenitors in the SVZ and the OSVZ are diverse in animals with cortical folds, this diversity may also be related to cortical folding (Reillo and Borrell, 2012; Betizeau et al., 2013; de Juan Romero et al., 2015; Johnson et al., 2015). Cortical folding has also been associated with the frequency of neural progenitor proliferation and gene expression patterns (Reillo et al., 2011; de Juan Romero et al., 2015; Toda et al., 2016; Matsumoto et al., 2017). Although many hypotheses had been proposed, experimental investigation of these hypotheses was delayed because of the difficulty of genetic manipulation in animals with cortical folds.

## Investigations of Cortical Folding Mechanisms Using Mice

Although the mouse cerebral cortex does not have cortical folds, there have been attempts to clarify the molecular mechanisms of cortical folding using mice. This is mainly because many genetic techniques, such as those used to make knockout mice or transgenic mice, are available. Early pioneering studies reported that enhancing the proliferation of neural progenitors could create a cortical fold-like curvature on the surface of the mouse cerebral cortex (Chenn and Walsh, 2002). Other studies reported that inhibition of cell death also produced curvatures of the surface of the cerebral cortex in mice (Haydar et al., 1999; Depaepe et al., 2005). Since then, introduction or knockout of various genes was reported to produce a cortical fold-like structure on the surface of the mouse cerebral cortex (Depaepe et al., 2005; Rash et al., 2013; Stahl et al., 2013; Florio et al., 2015; Ju et al., 2016; Martínez-Martínez et al., 2016; Wang et al., 2016; del Toro et al., 2017; Liu et al., 2017; Chizhikov et al., 2019; Shao et al., 2020; Han et al., 2021; Kerimoglu et al., 2021; Kyrousi et al., 2021; Shqirat et al., 2021; Wang et al., 2021). These results are intriguing because they experimentally demonstrated that curvature of the surface of the cerebral cortex can be produced by manipulating neural progenitors. It would be important to investigate the roles of these genes in cortical folding of the gyrencephalic brains, and ferrets would be an important option for these investigations.

## Features of Ferrets as an Experimental Model Animal

Ferrets are medium-sized carnivorous mammals with a body length of about 50 cm, a weight of 1–2 kg, and an average lifespan of 6–10 years (Figure 1A). They are thought to be a descendant of the European polecat and have been domesticated. Ferrets have the following advantages for investigating the mechanisms underlying the development and evolution of the cerebral cortex. First, by using ferrets, it is possible to analyze the mechanisms underlying the expansion and folding of the cerebral cortex and the amplification of oRG cells, as mentioned above. It would be intriguing to combine *in vivo* studies using ferrets and *in vitro* studies using human iPS/ES organoids to uncover the mechanisms that caused changes to the cerebral cortex during evolution. Second, because ferrets have been used for electrophysiological and neuroanatomical experiments, electrophysiological and anatomical information on the ferret brain is available (Cucchiaro and Guillery, 1984; Law et al., 1988; Sur et al., 1988; Hahm et al., 1991; Meister et al., 1991; Callaway and Katz, 1993; Mooney et al., 1993; Crowley and Katz, 2000; Borrell and Callaway, 2002; Huberman et al., 2003; Kawasaki et al., 2004). For example, many fundamental findings regarding neural plasticity and its critical period were discovered in studies of ocular dominance columns in the visual cortex and eye-specific segregation of retinogeniculate projections in the thalamus of the ferret visual system. This electrophysiological and anatomical information provides an important basis for interpreting the experimental results of genetic studies. Third, ferret pups are born in an immature state, making them suitable for analyzing developmental processes and molecular mechanisms. Cortical folding proceeds after birth in developing ferrets, whereas it is largely completed before birth in cynomolgus monkeys. Fourth, in addition to being used in neuroscience research, ferrets have been widely used in research in other fields, for example, in research on infectious diseases such as the influenza virus and on the mechanisms of vomiting and antiemetic reagents. As a result, they are easily obtained, and many researchers already have at least some familiarities with them. Finally, because knowledge regarding their breeding and mating has accumulated, ferrets are easy to raise. Despite these advantages, however, genetic techniques necessary for investigating the molecular mechanisms underlying the development and evolution of the ferret brain had been poorly available.

## Genetic Manipulation Techniques for the Ferret Brain

Because of the ferret’s advantages as a model animal, genetic techniques that could be applied to the ferret brain were desirable. One technique that was needed was genetic screening. Therefore, we made a custom ferret microarray for identifying genes expressed with characteristic patterns in the ferret brain (Kawasaki et al., 2004). Using this microarray, we have identified genes that are selectively expressed in magnocellular or parvocellular neurons, which are characteristically found in the well-developed visual system of higher mammals (Kawasaki et al., 2004; Iwai et al., 2013; Sato et al., 2017). Similarly, genes preferentially expressed in future gyral regions and future sulcal regions were uncovered using a ferret microarray (de Juan Romero et al., 2015). More recently, whole transcriptome RNA-seq analysis was applied to ferrets, and ferret RG cells were found to share key transcriptional features with human RG cells (Johnson et al., 2015). In addition, the ferret genome was sequenced, and annotated DNA sequence data is partially available (Peng et al., 2014). As these examples show, various genetic screening methods are now available for ferrets.

**FIGURE 3:**
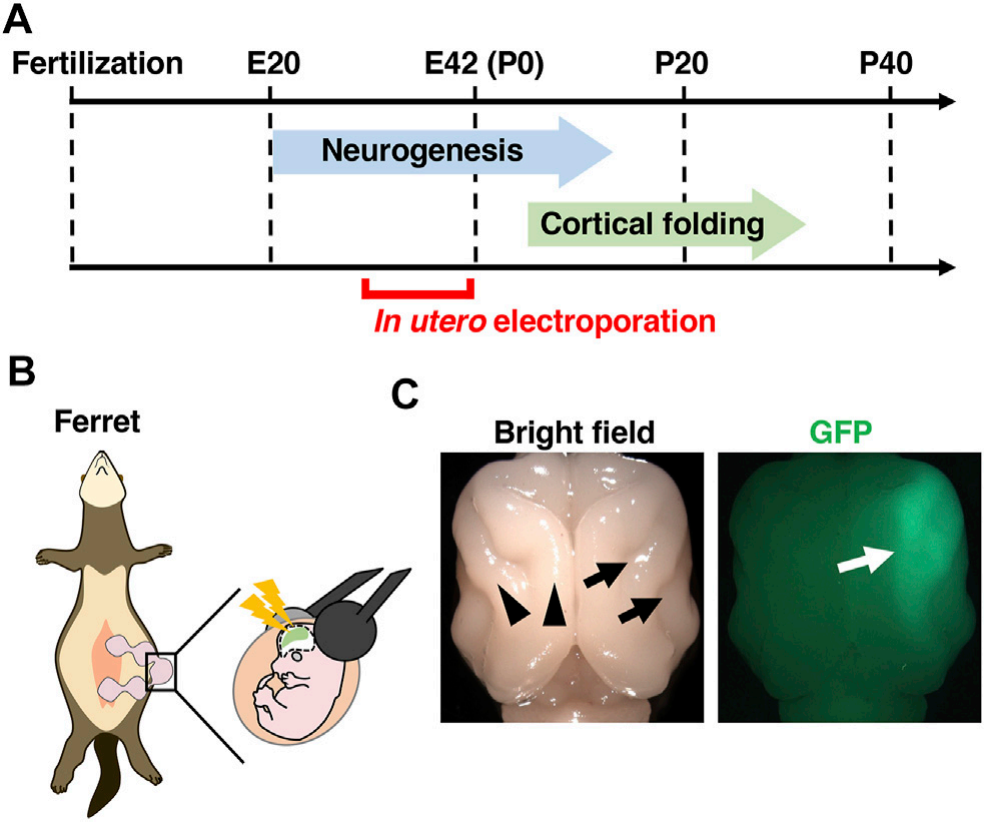
*In vivo* genetic manipulation for the ferret cerebral cortex using *in utero* electroporation. **(A)** A schema showing the developmental timing of neurogenesis and cortical folding, and the stages when *in utero* electroporation can be performed. Neurogenesis in the ferret cerebral cortex starts around embryonic day 20 (E20) and completes around postnatal day 1 (P1) in the somatosensory cortex and P14 in the visual cortex. Cortical folding of the ferret cerebral cortex starts around P4 and completes around P30. *In utero* electroporation of the ferret cerebral cortex can be performed from around E30, when layer 5/6 neurons are generated. **(B)** An illustration of an *in utero* electroporation procedure. **(C)** Dorsal views of a ferret brain in which GFP was expressed by *in utero* electroporation. Cortical folds, i.e. gyri (black arrowheads) and sulci (black arrows), are present on the cerebral cortex. GFP signals are observed on the right side of the cerebral cortex (white arrow).

Another important technique is genetic manipulation. Previous pioneering studies reported that transgenes can be transfected into the ferret brain using postnatal electroporation and *in vivo* retroviral vector injection (Borrell, 2010; Nonaka-Kinoshita et al., 2013). Aiming to create a convenient genetic manipulation technique that could be applied to most cortical neurons in the ferret cerebral cortex, we established an *in utero* electroporation technique for the ferret cerebral cortex (Figure 3) (Kawasaki et al., 2012; Kawasaki et al., 2013). Using this technique, not only most cortical neurons but also neural progenitors such as RG cells, IP cells and oRG cells can be transfected. It takes only 1 hour to perform the *in utero* electroporation procedure on one pregnant ferret mother, and transfected ferret babies are born within a few weeks after the procedure, allowing transfected ferrets to be easily and rapidly obtained. Furthermore, multiple kinds of plasmids can be co-transfected by just mixing them, and various plasmids can be applied to different embryos in the same ferret mother, making it possible to conduct experiments under many different conditions simultaneously using one ferret mother. We also succeeded in gene knockout in the ferret cerebral cortex by combining *in utero* electroporation and the genome editing technology CRISPR/Cas9 (Shinmyo et al., 2017). Transgenes can also be knocked-in in ferret cortical neurons using the CRISPR/Cas9 system (Tsunekawa et al., 2016). Another approach of genetic manipulation would be to create genetically modified ferrets. Knockout ferrets have been successfully created using genome editing techniques, and they were used to uncover the roles of Aspm and Disc1 in the ferret brain (Kou et al., 2015; Johnson et al., 2018). Transgenic ferrets were also made by inserting transgenes into the ROSA26 locus using the CRISPR/Cas9 system (Yu et al., 2019). Furthermore, because ferret iPS cells have been generated (Gao et al., 2020; Yoshimatsu et al., 2021), organoid research using ferret iPS cells would be feasible. Due to these technological developments, genetic analyses of the ferret brain have become increasingly popular.

## Investigation of the Mechanisms Underlying Neurogenesis and Cortical Folding Using Ferrets

### The Importance of Neural Progenitors in Cortical Folding

An increasing number of laboratories are using ferrets to analyze the molecular mechanisms of cortical folding. Pioneering studies reported the importance of neural progenitors in cortical folding. In the developing ferret cerebral cortex, pharmacological suppression of the proliferation of neural progenitors inhibited cortical folding (Haddad et al., 1979). Conversely, stimulation of neural progenitor proliferation in the developing ferret cerebral cortex promoted the formation of cortical folds (Nonaka-Kinoshita et al., 2013; Masuda et al., 2015). Importantly, even before cortical folds are formed in the developing cerebral cortex, the amount of cell proliferation is higher in future gyral regions, whereas it is lower in future sulcal regions (Reillo et al., 2011). Consistently, progenitors are more abundant in future gyral regions than in future sulcal regions in the embryonic monkey cerebral cortex (Smart et al., 2002). These results suggest that the increase in neural progenitors in future gyral regions is involved in cortical folding. However, even if the proliferation of neural progenitors is enhanced in the developing mouse cerebral cortex, cortical folds do not necessarily form (Nonaka-Kinoshita et al., 2013). Therefore, it seems that proliferation of neural progenitors is not the only factor mediating cortical folding.

To investigate which types of neural progenitors are important for cortical folding, the distribution of oRG and IP cells in the developing ferret cerebral cortex was analyzed. oRG and IP cells were unevenly distributed, being more abundant in areas that would become gyri, but less abundant in areas that would become sulci. This result raised the possibility that oRG cells and/or IP cells are more abundantly distributed in future gyral regions, and the greater number of neurons they provide in these regions cause outward protrusions that become gyri. Recently, we found that oRG cells in the developing ferret cerebral cortex can be subdivided into two groups, HOPX-positive and HOPX-negative, according to the presence or absence of the transcription factor HOPX (Matsumoto et al., 2020). The distribution of these cells suggests that HOPX-positive oRG cells are more likely to be involved in the formation of cortical folds. It is important to note that although oRG cells are less abundant in the developing mouse cerebral cortex (Shitamukai et al., 2011), an area with relatively many oRG cells was found in the medial region of the cerebral cortex, and this area seemed similar to the developing cerebral cortex of the gyrencephalic brain (Vaid et al., 2018). Furthermore, Hopx was shown to be important for increasing the number of oRG cells in the mouse cerebral cortex (Vaid et al., 2018). Therefore, HOPX might be a key regulator for the production of oRG cells in the gyrencephalic cerebral cortex.

### Mechanisms Regulating the Abundance of Neural Progenitors

A further important question is what the regulatory mechanisms upstream of neural progenitor proliferation are. To elucidate genes responsible for the proliferation of neural progenitors, we focused on human diseases which exhibit abnormal cortical folds.

Thanatophoric dysplasia is a congenital disease that shows abnormalities in bones and the brain including polymicrogyria, in which many more cortical folds are produced than normal. A pioneering study identified a mutation in the fibroblast growth factor (FGF) receptor 3 gene in polymicrogyria patients and showed that this mutation induced an activated form of the FGF receptor 3, raising the possibility that FGF signaling is involved in cortical folding and neural progenitor proliferation (Shiang et al., 1994). To directly test if FGF signaling mediates the proliferation of neural progenitors, FGF8, a ligand for the FGF receptor 3, was expressed in the developing ferret cerebral cortex using *in utero* electroporation (Masuda et al., 2015). The proliferation of oRG cells was promoted by the activation of FGF signaling, and as a result, oRG cells were increased. Consistently, when FGF signaling was inhibited by expressing a dominant-negative form of the FGF receptor 3 in the ferret cerebral cortex, the proliferation of oRG cells was reduced, and the number of oRG cells decreased (Matsumoto et al., 2017). These results indicate that FGF signaling regulates the proliferation of oRG cells.

**FIGURE 4:**
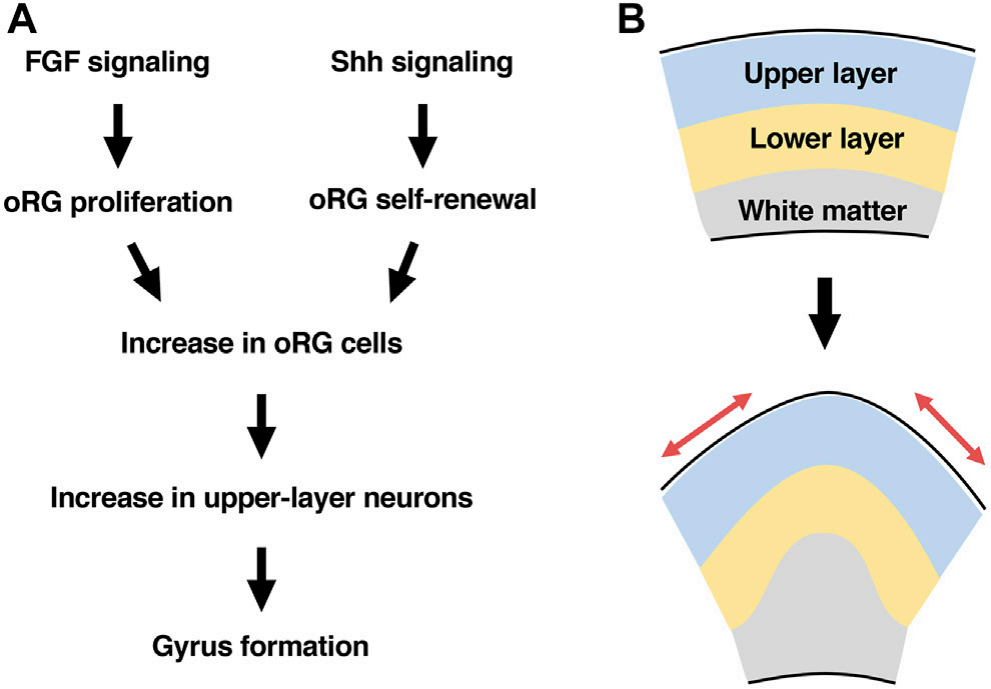
A hypothesis about mechanisms underlying cortical folding. **(A)** FGF signaling and Shh signaling cooperate to increase oRG cells by promoting proliferation and self-renewal, respectively. The increased oRG cells preferentially produce upper-layer neurons, resulting in gyrus formation. **(B)** Gyrus formation requires the tangential expansion of the cortical surface (red arrows), which is achieved by a preferential increase in upper-layer neurons.

We also investigated the roles of sonic hedgehog (Shh) signaling in the regulation of oRG cells because a previous report identified abnormality in Shh signaling in human patients with cortical fold malformations. When Shh ligand was introduced into the developing ferret cerebral cortex using *in utero* electroporation, the number of oRG cells was increased due to enhanced oRG cell self-renewal (Matsumoto et al., 2020). Consistently, Smoothened Agonist (SAG), a specific activator of Shh signaling, promoted self-renewal of oRG cells in cultured ferret brain slices (Hou et al., 2021). When Shh signaling was suppressed by introducing HhipΔC22, a dominant-negative form of the Shh signaling pathway, the number of oRG cells was decreased due to reduced oRG cell self-renewal. These findings suggest that FGF signaling and Shh signaling cooperate to increase oRG cells by promoting proliferation and self-renewal, respectively (Figure 4A) (Masuda et al., 2015; Matsumoto et al., 2017; Matsumoto et al., 2020).

Hippo signaling and serotonin signaling were also reported to be crucial for the proliferation and abundance of basal progenitors (Kostic et al., 2019; Xing et al., 2020). Moreover, when the human-specific gene ARHGAP11B was introduced into the ferret cerebral cortex, it increased oRG cells (Kalebic et al., 2018). Interestingly, PALMDELPHIN was reported to increase processes of basal progenitors and stimulate their proliferation through integrin signaling (Kalebic et al., 2019). It would be important to investigate the interactions among these signaling pathways to uncover the complete picture of the mechanisms regulating the proliferation and self-renewal of oRG cells.

In addition to elucidating the mechanisms of neural progenitor proliferation and self-renewal, uncovering those of the translocation of neural progenitors is also important. In a study comparing mice and ferrets, species-specific differences in interkinesis strategies were reported (Okamoto et al., 2014). As for the molecular mechanisms, Lzts1, which is associated with microtubule components, was reported to control the delamination and generation of oRG-like cells (Kawaue et al., 2019). Because few oRG cells are observed in mice, ferrets seem useful for examining the mechanisms underlying the translocation and morphological changes of oRG cells.

### Mechanisms Underlying Cortical Folding

Because both FGF signaling activation and Shh signaling activation increased oRG cells, we investigated whether FGF signaling and Shh signaling are involved in cortical folding (Masuda et al., 2015; Matsumoto et al., 2017; Matsumoto et al., 2020). When FGF signaling was activated by introducing FGF ligand into the developing ferret cerebral cortex, the number of cortical folds was increased, resulting in a polymicrogyria-like phenotype. Importantly, the increased cortical folds exhibited the critical features of physiological cortical folds. These folds contained all cortical layers (i.e. layers 1–6), and while the cortical surface exhibited additional cortical folds, the ventricular surface was smooth and without curvature. Furthermore, inhibiting FGF signaling by expressing a dominant-negative form of the FGF receptor in the developing ferret cerebral cortex attenuated cortical folding. Similarly, when Shh signaling was activated by introducing Shh ligand into the developing ferret cerebral cortex, the number of cortical folds was increased. Suppression of Shh signaling using HhipΔC22 inhibited cortical folding. These results indicate that FGF signaling and Shh signaling cooperate to induce cortical folds, presumably through regulating the number of oRG cells (Figure 4A) (Masuda et al., 2015; Matsumoto et al., 2017; Matsumoto et al., 2020).

Interestingly, when comparing mice and ferrets, the amount of Shh protein in the cerebral cortex was found to be higher in ferrets (Matsumoto et al., 2020). Furthermore, Gli1 expression levels, which reflect the activation of Shh signaling, were higher in the cerebral cortex of ferrets than in that of mice. These results indicate that Shh signaling is more strongly activated in the ferret cerebral cortex than in the mouse cerebral cortex and may indicate that increased Shh signaling activity during evolution led to an increase in oRG cells and the acquisition of cortical folds (Matsumoto et al., 2020).

A further important question was what mechanisms link an increase in oRG cells to the morphological changes leading to cortical folds. In detailed studies of the cerebral cortex in which FGF signaling or Shh signaling was activated to promote cortical folding, the thickness of superficial layers of the cerebral cortex was selectively increased, while deep layers were less affected (Masuda et al., 2015; Matsumoto et al., 2017; Matsumoto et al., 2020). This result is consistent with the previous hypothesis that the ratio between superficial and deep regions of the cerebral cortex is important for cortical folding (Richman et al., 1975; Kriegstein et al., 2006). In order to test this hypothesis, Cdk5 was used to selectively reduce the number of neurons in superficial layers of the cerebral cortex. Cdk5 has been reported to be responsible for human lissencephaly (Magen et al., 2015) and therefore is thought to be important for cortical folding. Consistent with the data from human lissencephaly patients, knocking out the Cdk5 gene in pyramidal neurons of the developing ferret cerebral cortex by combining *in utero* electroporation and the CRISPR/Cas9 system attenuated cortical folding, suggesting that Cdk5 in pyramidal neurons is crucial for cortical folding (Shinmyo et al., 2017). Cdk5 is required for radial migration of neurons from the ventricular surface to the brain surface, suggesting that radial migration of cortical neurons is crucial for cortical folding. Consistently, introduction of a mutated SCN3A/Na_v_1.3 sodium channel into cortical neurons inhibited both radial migration and cortical folding (Smith et al., 2018). In order to determine to which layers of the cerebral cortex it is important for neurons to migrate, a dominant-negative form of Cdk5 was selectively introduced into either layer 2/3 neurons or layer 5–6 neurons. Interestingly, suppressing the radial migration of layer 2/3 neurons significantly inhibited cortical folding, whereas suppressing that of layer 5–6 neurons did not. This result supports the hypothesis that a preferential increase in superficial regions of the cerebral cortex relative to deep regions induces cortical folding (Figures 4A,B). It should be noted that cortical neurons migrate in a tangential orientation without following strict radial paths in the developing ferret cerebral cortex (Reillo et al., 2011; Gertz and Kriegstein, 2015). Therefore, it seems plausible that cortical folding requires a tangential expansion of the superficial portion of the cerebral cortex.

As mentioned above, the mechanisms of cortical folding have been intensively investigated. However, there are still many aspects that are not yet clearly understood. First, the formation of cortical folds continues even after neurons have completed their neurogenesis and radial migration during development. This suggests that mechanisms other than the proliferation of neural progenitors and radial migration of cortical neurons are also involved in cortical folding. In addition to intrinsic genetic factors, some non-genetic factors could be involved in cortical folding because patterns of cortical folds are not completely identical even between genetically identical twins (Lohmann et al., 1999). Since cortical folding proceeds after birth in ferrets, the ferret may be a useful model to study the influence of not only intrinsic genetic factors but also extrinsic environmental factors such as birth.

### Common and Species-specific Mechanisms Underlying Cortical Folding

An important question is whether the genetic programs controlling cortical folding found in ferrets are conserved in primates. Folds of the cerebral cortex are present in many mammalian orders, while they are absent in some species including mice. It seems likely that gyrencephalic animals such as humans and ferrets, at least in part, share common mechanisms regulating cortical folds, although ferrets are phylogenetically farther from primates than rodents. Since FGF signaling, Shh signaling and Cdk5 are involved in cortical folding in both humans and ferrets, it is plausible that the regulation of oRG cell amplification by FGF signaling and Shh signaling is conserved in both species. This idea is also supported by numerous previous studies showing that neural progenitors in gyrencephalic animals share common features that are lacking in those in mice. For example, larger amounts of neural progenitors are seen in the OSVZ of various gyrencephalic animals including humans, monkeys, ferrets and guinea pigs (Kriegstein et al., 2006; Martínez-Cerdeño et al., 2006; Hansen et al., 2010; Reillo and Borrell, 2012; Dehay et al., 2015; Hatakeyama et al., 2017). Moreover, differentially expressed genes between prospective gyri and sulci in neural progenitors of the ferret cerebral cortex exhibit a similar expression pattern to those in the developing human cerebral cortex (de Juan Romero et al., 2015). It was recently shown that microRNA miR-3607 plays a key role in the amplification of RG cells by acting as a regulator of Wnt/β-catenin signaling in ferrets and humans and that the loss of miR-3607 expression during evolution reduced the amplification of neural progenitors in the mouse cerebral cortex (Chinnappa et al., 2022). It seems possible that the common ancestor of mammals had folds of the cerebral cortex and that mice underwent a secondary loss of cortical folds (Kelava et al., 2013).

It also should be noted that although ferrets share several developed brain structures with humans, these structures in humans are often more developed than those in ferrets. Indeed, in addition to brain size, the gyrification index, which indicates the extent of cortical folds, and the thickness of superficial layers of the cerebral cortex, are larger in humans (Hutsler et al., 2005; Zilles et al., 2013). These facts suggest the emergence of primate-specific mechanisms that enhanced brain growth and cortical folding. Recent studies have identified primate-specific and human-specific genes and noncoding microRNAs that promote the amplification of neural progenitors (Arcila et al., 2014; Florio et al., 2015; Fiddes et al., 2018; Florio et al., 2018; Nowakowski et al., 2018; Suzuki et al., 2018). It was demonstrated that introduction of the human-specific gene ARHGAP11B in the ferret brain resulted in a further expansion of the cerebral cortex (Kalebic et al., 2018). Thus, the ferret is an important model organism for the investigation of common mechanisms of cortical folding among gyrencephalic animals as well as the impact of primate-specific and human-specific genomic changes on brain growth and cortical folding in the gyrencephalic cerebral cortex.

## Evolution and Development of the Fiber Layer in the Cerebral Cortex

**FIGURE 5:**
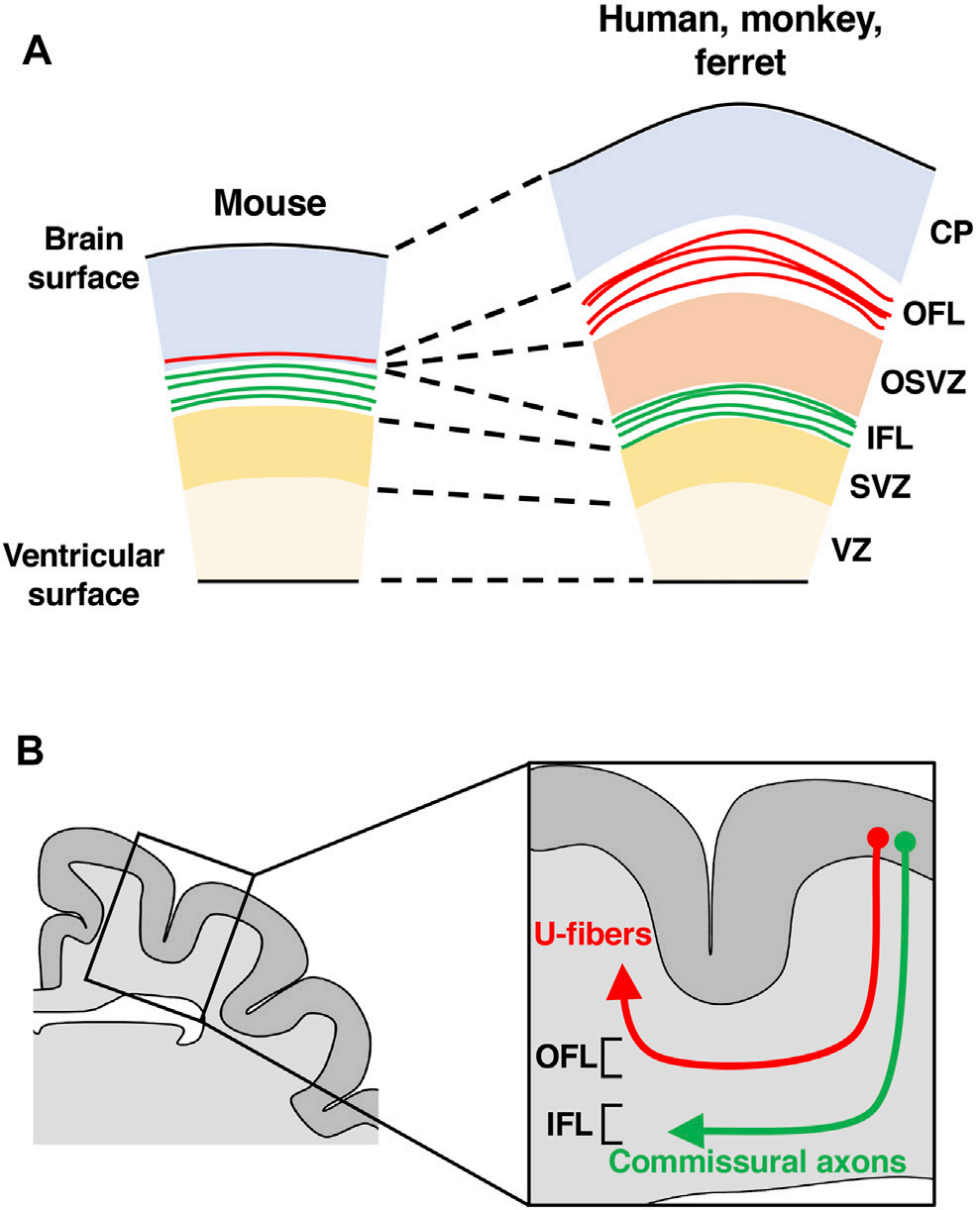
A hypothesis about the evolution of fiber layers in the developing cerebral cortex. **(A)** Schemas of cross sections of the developing cerebral cortices of mice, ferrets, monkeys and humans. Fibers in the outer fiber layer (OFL, red) increased during evolution. **(B)** A schema of a cross section of the ferret cerebral cortex. The OFL becomes subcortical U-fibers (red), while the IFL gives rise to commissural axons (green) and subcortical projecting axons. CP, cortical plate; OSVZ, outer subventricular zone; SVZ, subventricular zone; VZ, ventricular zone.

In the developing cerebral cortex of humans and monkeys, in addition to the cortical plate and the germinal zones, there are two fiber layers, the inner fiber layer (IFL) and the outer fiber layer (OFL) (Figure 5A) (Molnár and Clowry, 2012). However, a detailed understanding of their development and evolution had remained elusive, at least partially because these two fiber layers were not recognized in the mouse cerebral cortex. Interestingly, when GFP was expressed in excitatory cortical neurons of the ferret cerebral cortex using *in utero* electroporation, GFP-positive axons were found to be accumulated in positions corresponding to where the IFL and the OFL are observed in humans and monkeys (Figure 5A) (Saito et al., 2019). These results suggest that ferrets, like humans and monkeys, also have the IFL and the OFL in the developing cerebral cortex and that the IFL and the OFL contain axons derived from excitatory neurons of the cerebral cortex (Saito et al., 2019).

To investigate which neuronal circuits the OFL and the IFL become after developmental processes proceed, we expressed GFP in cortical neurons and examined the projections of GFP-positive axons corresponding to the IFL and the OFL. The IFL was found to give rise to mainly commissural and subcortical projecting axons, whereas the OFL became U-fibers (Figure 5B) (Yoshino et al., 2020). U-fibers are short association fibers located just below gray matter and have been found in humans and monkeys (Meynert, 1885; Nieuwenhuys et al., 1988; Schuz et al., 2002; Catani et al., 2012; Ouyang et al., 2017). U-fibers are thought to be important for functional association between neighboring cortical areas, and their abnormalities have been reported in neurodevelopmental and psychiatric disorders. MRI and histological analyses of U-fibers have been performed on human and monkey brains, but fibers corresponding to the IFL and the OFL have not been reported in mice, making an investigation of U-fibers difficult. Taken together, these results suggest that ferrets are a useful model organism for investigating U-fibers (Yoshino et al., 2020).

Because U-fibers can be visualized in ferrets by expressing GFP using *in utero* electroporation, we performed similar experiments using mice. Interestingly, a small number of GFP-positive axons that project to neighboring cortical areas were observed in mice (Saito et al., 2019; Yoshino et al., 2020). This result suggests that a small number of axon fibers corresponding to U-fibers found in humans, monkeys and ferrets also exist in mice, and these axon fibers have increased significantly during evolution, forming the OFL (Figure 5A, red) (Saito et al., 2019; Yoshino et al., 2020). Although these results have clarified some aspects of the development and evolution of U-fibers, many points still remain unclear. It would be important to elucidate the molecular mechanisms that regulate the formation of U-fibers and the physiological significance and pathological involvement of U-fibers. Furthermore, because U-fibers are predominantly observed in the gyrencephalic mammalian cerebral cortex, it would be intriguing to investigate the roles of U-fibers in cortical folding.

## Future Prospects

The mammalian cerebral cortex continues to be of great interest to researchers. Our understanding of the developmental processes, functions and diseases of the cerebral cortex has been advanced mainly using mice as a model animal. Recently, the use of new animal models such as ferrets and marmosets, along with the development of various genetic techniques for them including *in utero* electroporation, genome editing and iPS/ES organoids, have expanded the scope of analyses of the cerebral cortex. It is expected that the use of new animal models will accelerate our understanding of the mechanisms underlying the development and evolution of the complex brain architecture and brain functions observed in the well-developed cerebral cortex of higher mammals, as well as those underlying the pathophysiology of diseases of the cerebral cortex. Ferrets should provide an important platform for investigating these mechanisms *in vivo*.
